# Effect of litter separation on 24-hour rhythmicity of plasma prolactin, follicle-stimulating hormone and luteinizing hormone levels in lactating rabbit does

**DOI:** 10.1186/1740-3391-3-9

**Published:** 2005-06-02

**Authors:** Pilar Cano, Vanessa Jiménez, Maria P Álvarez, Mario Alvariño, Daniel P Cardinali, Ana I Esquifino

**Affiliations:** 1 Departamento de Bioquímica y Biología Molecular III, Facultad de Medicina, Universidad Complutense de Madrid, 28040 Madrid, Spain; 2 Departamento de Biología Celular, Facultad de Medicina, Universidad Complutense de Madrid, 28040 Madrid, Spain; 3 Departamento de Producción Animal, E.T.S.I. Agrónomos, Universidad Politécnica de Madrid, Spain; 4 Departamento de Fisiología, Facultad de Medicina, Universidad de Buenos Aires, 1121 Buenos Aires, Argentina

## Abstract

**Background:**

This work describes the effect of a 48-h litter separation on 24-h patterns of plasma prolactin, FSH and LH concentration in female lactating rabbits kept under a 16:8 light-dark photoperiod (lights on at 0800 h).

**Methods:**

Groups of 6–7 female lactating rabbits maintained with their litters or separated from them for 48 h were killed by decapitation on day 11 post-partum, at 6 different time points throughout a 24-h cycle, starting at 0900 h. Plasma levels of prolactin, FSH and LH were measured by specific double antibody radio-immunoassays.

**Results:**

Plasma level of prolactin in control and separated does changed in a similar way throughout the day, showing two maxima, at 0500–0900 h and at 1700–2100 h, respectively. Litter separation significantly augmented plasma FSH and LH and disrupted their 24-h rhythmicity.

**Conclusion:**

Since previous studies had shown that litter separation for short periods of time augmented sexual receptivity and fertility of the doe, the changes in FSH and LH reported may influence the massive release of gonadotropin releasing hormone, LH and FSH triggered by mating or artificial insemination in litter-separated mothers.

## Introduction

In nursing rabbits, sexual receptivity and fertility achieved after artificial insemination is depressed during the period of lactation, presumably through a hormonal antagonism between prolactin and gonadotropin release [1-3]. Several studies have demonstrated that separation of the doe from its litter for short periods of time, prior to artificial insemination, is very effective in stimulating ovarian activity in the mother [4-6], with endocrine changes that may explain the activation of ovarian function [7-9] Thus, separation of the does from their litters could be an effective procedure to augment breeding efficiency under farm conditions [5-7].

It must be noted that the published studies on the endocrine changes taking place in the doe after litter separation have been performed at single time points in the daily cycle, usually in the morning, which is an important drawback in view of the circadian nature of the secretion of the pituitary hormones involved [10,11]. Indeed a number of circadian functions have been examined in rabbits [12-17], but there is no information on 24-h pattern of hormone release. This prompted us to undertake the present study whose aim was to examine the effect of litter separation for 48 h on 24-h changes in plasma prolactin (PRL), follicle stimulating hormone (FSH) and luteinizing hormone (LH) levels of the doe. Specifically, we sought to answer two questions: (i) did suppression of a major neuroendocrine and circadian stimulus like the stimulation of nipples by the lactating pup affect the 24-h changes in gonadotropin and prolactin release?; (ii) could the changes in circulating hormone levels be related to augmentation of breeding efficiency found after litter separation from the doe?

## Materials and methods

### Animals

The study was performed in 84 multiparous, lactating Californian x New Zealand White crossbreed female doe rabbits. Animals were housed in the research facilities of the Animal Production Department, Universidad Politécnica de Madrid, under controlled light-dark cycles (LD 16:8, light on at 8:00 h), housed in individual metal cages, fed at libitum using a commercial pellet diet (Lab Rabbit Chow, Purina Mills, Torrejón de Ardoz, Madrid, Spain) and having access to tap water ad libitum. The study was performed according to the CEE Council Directives (86/609, 1986) for the care of experimental animals. Groups of 6–7 female lactating rabbits were maintained with their litters or separated from them for 48 h, starting at different times (i.e., at 09:00, 13:00, 17:00, 21:00, 01:00 or 05:00 h). Ninety five percent of the does suckled the pups between 03:30 and 04:30 h during the dark period, as has been previously described [16]. On day 11 post-partum, the does were killed by decapitation at 6 different time points throughout a 24-hour cycle starting at 0900 h. Blood was collected from the cervical wound and the plasma was separated to measure prolactin and gonadotropin concentration.

### Hormone assays

Plasma prolactin, FSH and LH levels were measured in duplicate samples by specific RIA methods [18] using AFP-991086, AFP-472176 and AFP-3120489 antibodies for prolactin, FSH and LH respectively, supplied by the National Institute of Health (NIH, Bethesda, MD, USA) and Dr. A. F. Parlow (Harbour-UCLA Medical Center, CA, USA). Hormones were labeled with ^125^I by the Chloramine T-method [19]. The antibody titers used were 1:62,500 for prolactin, 1:45,000 for FSH and 1:250,000 for LH assays, respectively. The volume of plasma used was 10 μl (prolactin assay), 75 μl (FSH assay) and 100 μl (LH assay). *Staphylococcus aureus *(prepared by the Department of Plant Physiology, U.A.M., Madrid, Spain) was used to precipitate the bound fraction [18]. The assays were previously validated in our laboratory [18]. All samples were measured in the same assay run to avoid inter-assay variations. The limits of detection for prolactin, FSH and LH were 0.125, 0.48 and 0.05 ng/mL respectively. The intra-assay coefficient of variation, calculated from a pool of plasma measured ten times in the same assay, was < 5%.

### Statistical analysis

After determining that the homogeneity-of-variance assumption was tenable and that the distribution appeared unimodal and nonskewed, the statistical analysis of results was performed by a two-way factorial analysis of variance (ANOVA). Generally, the factorial ANOVA included assessment of the group effect (i.e. the occurrence of differences in mean values between control and separated groups), of time of day effects (the occurrence of daily changes) and of the interaction between the two factors (separation and time, from which inference about differences in timing and amplitude could be obtained). A one-way ANOVA followed by Student-Newman-Keuls' test was then employed to show which time points were significantly different within each experimental group to define the existence of peaks. A Student's t test was performed to assess differences between the experimental groups at particular time intervals. P values lower than 0.05 were considered evidence for statistical significance.

## Results

**Figure 1:**
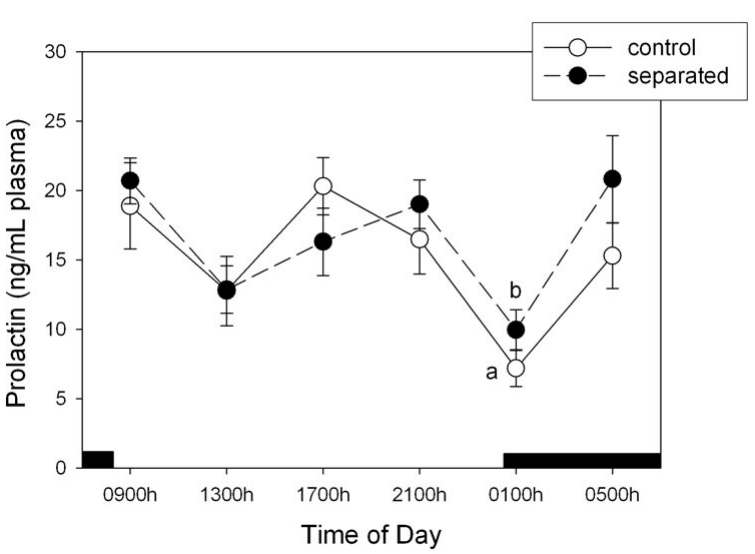
**24-h changes in plasma prolactin levels in female lactating rabbits**. Groups of 6–7 animals maintained with their litters or separated from them for 48 h were killed by decapitation on day 11 post-partum, at 6 different time points throughout a 24-h cycle, starting at 0900 h. The dark bar indicates scotophase duration. Results are the means ± SEM. Letters indicate the existence of significant differences between time points in each group after a one-way ANOVA followed by a Student-Newman-Keuls' test, as follows: ^a^p < 0.05 vs. 09:00 and 17:00 h, ^b^p < 0.01 vs. 09:00 h, p < 0.05 vs 05:00 h. For further statistical analysis, see text.

Figure 1 depicts the plasma prolactin levels through a 24-h cycle in control does and in does separated from their litters for 48 h. Analyzed as a main factor in a factorial ANOVA, significant time of day changes occurred (F = 35.4, p < 0.00001) with absence of any significant effect of litter separation. Both in control and separated does, plasma level of prolactin changed throughout the day showing two maxima, at 0500 – 0900 h and at 1700 – 2100 h, respectively (p < 0.001, Figure 1).

**Figure 2:**
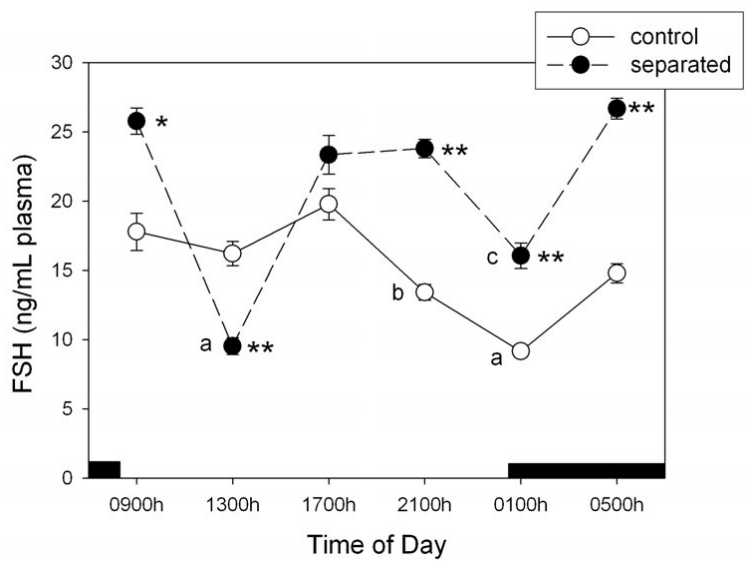
**24-h changes in plasma FSH levels in female lactating rabbits**. Groups of 6–7 animals maintained with their litters or separated from thrm for 48 h were killed by decapitation on day 11 post-partum, at 6 different time points throughout a 24-h cycle, starting at 0900 h. The dark bar indicates scotophase duration. Results are the means ± SEM. Asterisks indicate significance differences with control at that particular time interval (Student's t test, * p < 0.05, ** p < 0.01). Letters indicate the existence of significant differences between time points in each group after a one-way ANOVA followed by a Student-Newman-Keuls' test, as follows: ^a^p < 0.01 vs. all remaining groups, ^b^p < 0.05 vs. 17:00 h, ^c^p < 0.01, vs 09:00, 13:00, 21:00 and 05:00 h, p < 0.05 vs. 17:00 h. For further statistical analysis, see text.

Figure 2 shows the 24-h changes in plasma FSH concentration in control does and in does separated from their litters for 48 h. Analyzed as a main factor in a factorial ANOVA, litter separation augmented FSH levels by 37 % (F = 104.6, p < 0.00001). A significant effect of time of day and a significant interaction "time of day x litter separation" were found (F = 41.1 and 23.3, p < 0.00001, respectively), i.e., a single maximum in the first half of the light period was seen in controls whereas two maxima, at 0500 – 0900 h and at 1700 – 2100 h, respectively, were found after litter separation.

**Figure 3:**
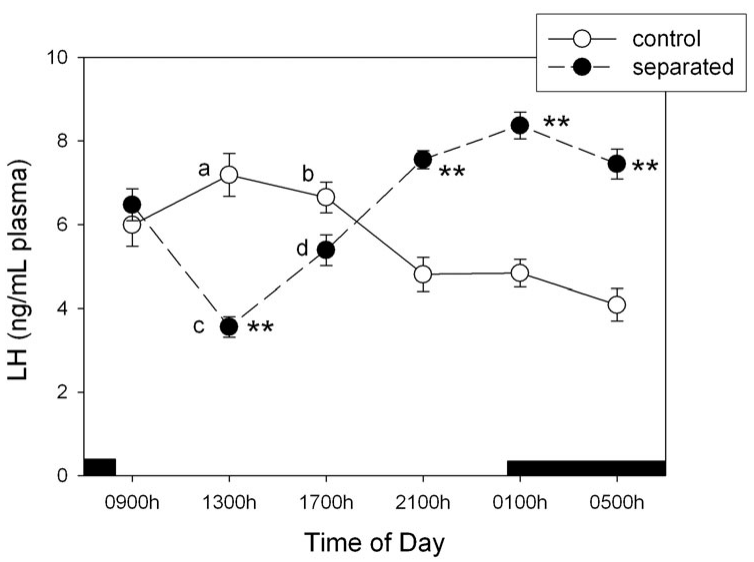
**24-h changes in plasma LH levels in female lactating rabbits**. Groups of 6–7 animals maintained with their litters or separated from them for 48 h were killed by decapitation on day 11 post-partum, at 6 different time points throughout a 24-h cycle, starting at 0900 h. The dark bar indicates scotophase duration. Results are the means ± SEM. Asterisks indicate significance differences with control at that particular time interval (Student's t test, * p < 0.05, ** p < 0.01). Letters indicate the existence of significant differences between time points in each group after a one-way ANOVA followed by a Student-Newman-Keuls' test, as follows: ^a^p < 0.05 vs 21:00, 01:00 and 05:00 h, ^b^p < 0.05, vs 05:00 h, ^c^p < 0.01 vs. 09:00, 21:00, 01:00 and 05:00 h, ^d^p < 0.05 vs. 21:00, 01:00 and 05:00 h. For further statistical analysis, see text.

Figure 3 displays the 24-h changes in plasma LH concentrations. A significant effect of litter separation and time of day was apparent (F = 16.1, p < 0.0001 and F = 2.51, p < 0.03, factorial ANOVA). Litter separation brought about a small albeit significant 16% increase in mean circulating LH values. As shown by the significant interaction "time of day x litter separation" found (F = 29.4, p< 0.00001), litter separation disrupted the plasma LH rhythm by phase-shifting its maximum by 12 h, from 1300 h in controls to 0100 h in separated mothers (Figure 3).

## Discussion

The questions posed in the Introduction may now be answered. First, our results indicate that the 24 h patterns of plasma FSH and LH, but not of prolactin, changed significantly in nursing rabbits after litter separation for 48 h. Second, litter separation disrupted the 24-h rhythmicity of plasma FSH and LH concentration and caused a moderate increase in their concentration (when assessed as the mean 24-h values). Previous studies in the rabbit showed that litter separation for short periods of time augmented sexual receptivity and fertility of the doe [4-9]. Thus, the changes in gonadotropins reported herein could be a reflection of the same mechanisms involved in the massive release of gonadotropin releasing hormone (GnRH), LH and FSH triggered by mating or artificial insemination in litter-separated mothers.

The rabbit exhibits an unusual form of maternal care, with a single and very short visit (3–5 min) every day to nurse [20]. This daily nursing visit of the doe is extremely regular, with some individuals showing a day-to-day variability of only a few minutes. Estrogen, androgen, progesterone and prolactin promote the onset of this behavior in does [21] while its maintenance relies on stimuli from the litter (i.e., maternal responsiveness is altered or abolished by prevention of mother/young contact at parturition or during early lactation). From a number of studies on the distribution of estrogen, androgen and prolactin receptors, quantification of expression of immediate-early genes, and lesions of structures of the olfactory system, it was concluded that rabbits rely on the same hormonal and extrahormonal factors that stimulate maternal behavior in other mammals except for the very peculiar circadian nursing pattern that is unique to rabbits [22-24].

Since the early observations by McNeilly and Friesen [25] it is known that postpartum blood levels of prolactin are similar in lactating and postpartum nonlactating females. Such an observation was confirmed in the present study in which plasma prolactin levels, measured at six time intervals in a 24-h cycle (the closest to nursing time was at 05:00 h), were essentially similar in control and litter-separated does. In lactating females, suckling evoked an immediate increase (3- to 5-fold) in circulating prolactin levels, an effect mimicked by the tactile stimulation of the teats [25]. Likewise, in hares, prolactin levels increased significantly during lactation only after suckling stimuli [26]. It must be noted that, in contrast to rabbits, plasma prolactin levels are significantly changed by nursing in most species, the suckling stimulus being an effective masking signal for the 24-h release of prolactin [27,28]. This does not occur in the doe, the circadian changes of plasma prolactin levels remaining essentially unchanged after litter separation (presumably because of the very short nursing period). This suggests that the circadian secretion of prolactin and the prolactin response to physical stimulation of the nipples are independent phenomena that occur throughout the nursing period.

Previous reports using single sampling procedures [8] indicated that litter separation decreased thr doe's prolactin levels and did not affect FSH. Discrepancies are possibly dependent on the sampling frequency and time of day examined. Collectively, the results underline the importance of performing 24-h studies to have a more precise picture of the hormonal changes.

Litter separation disrupted the 24-h rhythmicity of both FSH and LH significantly. McNeilly [29] suggested that a reduction of plasma LH levels found during the light period could be coupled to an increase of pulsatile pattern of hypothalamic GnRH release. In the present study, the does exhibited, after litter separation, an inverse 24-h pattern of LH release with the lowest values during the light phase of daily photoperiod. Presumably, the disrupted 24-h rhythmicity of LH (and FSH) are linked to the greater mating or artificial insemination-induced release of LH and FSH found in does separated from their litters.

In summary, the present study demonstrates the existence of 24-h variations in circulating prolactin, LH and FSH levels in nursing does. Litter separation for short periods of time, an effective procedure to stimulate ovarian activity prior to artificial insemination, markedly influences the secretory patterns of FSH and LH, a finding that can be related to the higher reflex ovulatory response to mating or artificial insemination observed in does separated from their pups. The specific value of the present study in terms of augmenting breeding efficiency should be further explored.
